# A comprehensive and perspective view of oncoprotein SET in cancer

**DOI:** 10.1002/cam4.1526

**Published:** 2018-05-10

**Authors:** Buuvee Bayarkhangai, Suzan Noureldin, Liting Yu, Na Zhao, Yaru Gu, Hanmei Xu, Changying Guo

**Affiliations:** ^1^ State Key of Natural Medicine China Pharmaceutical University Nanjing China

**Keywords:** cancer therapy, function, oncoprotein SET, regulation

## Abstract

SET is a multifunctional oncoprotein which is ubiquitously expressed in all kinds of cells. The SET protein participates in many cellular processes including cell cycle, cell migration, apoptosis, transcription, and DNA repair. Accumulating evidence demonstrates that the expression and activity of SET correlate with cancer occurrence, metastasis, and prognosis. Therefore, the SET protein is regarded as a potential target for cancer therapy and several inhibitors are being developed for clinical use. Herein, we comprehensively review the physiological and pathological functions of SET as well as its structure‐function relationship. Additionally, the regulatory mechanisms of SET at both transcriptional and posttranslational levels are also discussed.

## 1 INTRODUCTION

SET, also known as template activating factor‐I β (TAF‐I β), inhibitor 2 of protein phosphatase 2A (I2PP2A), and putative histocompatibility leukocyte antigen class II‐associated protein II (PHAPII), is a multifunctional oncoprotein which is involved in many cellular processes. It was firstly identified as the *set‐can* fusion gene in an acute undifferentiated leukemia patient in 1992.^1^, ^2^ Study of this fusion gene has unveiled the connection between SET and cancer, and a subset of these studies has demonstrated that SET plays an important role in tumorigenesis and metastasis. SET is ubiquitously expressed in many types of human tissues including kidney, liver, brain, spleen, lung, heart, and the gonadal system.^3^ Due to its role in cellular functions, its dysregulation, especially overexpression, contributes to the development of various diseases including Alzheimer's disease, polycystic ovary syndrome, and different types of cancer.^4^, ^5^, ^6^, ^7^, ^8^ Recently significant progress has been made in understanding the physiological and pathological functions of SET. In this context, we will provide the latest and comprehensive overview of SET in cancer including characterization of SET and its homologs, its functional roles and underlying mechanisms and transcriptional and post‐translational regulation as well as its potential as a therapeutic target.

## 2 UPREGULATION OF THE ONCOPROTEIN SET IN CANCER

Accumulating evidence has demonstrated upregulation of the oncoprotein SET in different types of cancer and its association with poor clinical outcomes. Overexpression of SET protein in hemopoietic cells always results in malignancies. For example, in chronic myeloid leukemia (CML) cells, SET protein was overexpressed and further upregulated during blast crisis.^9^ A similar phenomenon was observed in B‐cell chronic lymphocytic leukemia (CLL), non‐Hodgkin lymphoma (NHL), and acute myeloid leukemia (AML) at both mRNA and protein levels. At the same time, increased SET protein levels correlated with worse clinical outcomes.^10^, ^11^ Studies on tumor samples from patients with hepatocellular carcinoma, pancreatic cancer, and metastatic colorectal cancer, respectively, verified that SET overexpression is tumor specific and contributes to tumor progression.^12^, ^13^, ^14^ Janghorban et al^15^ found that about 50% of breast cancer cell lines showed overexpression of SET by RNA‐seq and Western blotting across all tumor subtypes, which was further confirmed using primary human breast tumor samples with patient‐matched adjacent normal tissue. SET deficiency significantly impaired the tumorigenic potential of breast cancer cell lines. SET is also reported to be upregulated in several other neoplasms at both the mRNA and protein levels, including head and neck squamous cell carcinoma, choriocarcinoma, Wilm's tumor, malignant brain tumors, alveolar soft part sarcoma, gastric carcinoma, testicular carcinoma, cancer of the breast, lung, liver, colon, and prostate. These data indicate that SET oncoprotein plays a fundamental role in tumorigenesis.^15^, ^16^, ^17^, ^18^

## 3 STRUCTURE AND SUBCELLULAR LOCATION

**Figure 1:**
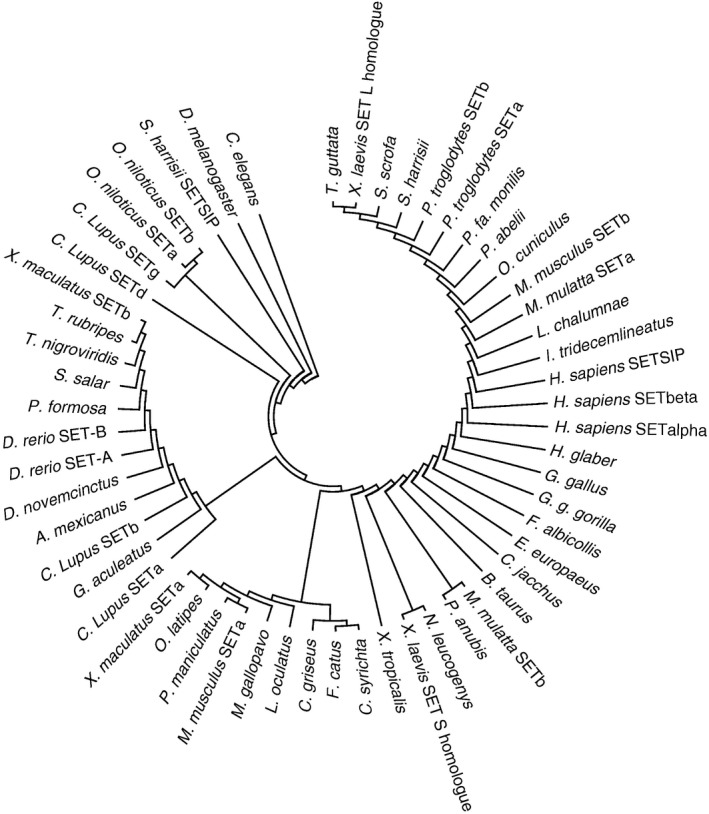
Phylogenetic analysis of SET protein in different species. The phylogenetic tree was constructed using the neighbor‐joining method, and evolutionary distances were computed using the p‐distance method. Evolutionary analyses were conducted using MEGA7.

From *Caenorhabditis elegans* to *Homo sapiens*, SET oncogenes are highly conserved during evolution (Figure 1). The human SET gene consists of 8 exons and is located on the q34.11 arm of the chromosome 9.^2^ There are 2 main SET isoforms: SET‐α and SET‐β (also referred as SET). Alternative promoter regions are responsible for different SET isoform transcripts.^3^ Additionally, SETSIP (SET similar protein) gene, located on chromosome 1 is believed to be a SET retrogene, which has 1 exon showing 94% homologous with SET‐α and 87% with SET‐β. SET protein is a structural homolog of nucleosome assembly proteins (NAPs) which participate in assembly/disassembly of the nucleosome as histone chaperones.^19^ SET protein has 3 distinct structural domains: N‐terminal region (1‐37/24), NAP domain (25‐225), and C‐terminal tail (226‐277). The crystal structure showed that SET protein forms a dimer structure with “headphone”‐like shape, and each subunit consists of an N‐terminal region with super‐coiled structure (1‐24), a backbone helix (25‐78) and an “earmuff” domain composed of 6 α‐helixes and 4 β‐sheets (79‐225).^19^ The C‐terminal tail (226‐277) was not able to be crystallized due to its highly soluble nature and acidic properties. Different domains of the SET protein are engaged in different cellular activities: Backbone helix is responsible for nucleosome assembly, the bottom half of the earmuff domain is necessary for histone and DNA binding, and acidic residues in the C‐terminus are able to interact with specific molecules, such as p53 and Ku70.^20^, ^21^

SET was initially reported as a predominant nuclear phosphoprotein.^2^ Further studies have revealed that SET distributes in the plasma membrane, cytoplasm, and nucleus.^6^, ^22^, ^23^, ^24^ Mouse SET protein localizes in both the cytoplasm and nucleus of most tissues including brain, kidney, liver, lung, and heart.^3^ Lieberman's laboratory demonstrated that SET localizes at the perinuclear region and endoplasmic reticulum in K562 cells. Interestingly, they found that harsh fixation conditions in the immunostaining procedure caused SET nuclear translocation which may explain why some published immunohistochemical studies identified SET as an exclusively nuclear protein.^25^ The shuttle of SET is controlled by an impα3/impβ‐dependent pathway.^24^ SET can interact with impα3 through the known nuclear localization signal (NLS) ^168^KRSSQTQNKASRKR^181^. Impα3 then recruits impβ to form a ternary nuclear pore complex, resulting in the efficient transportation of SET into the nucleus. Additionally, the sequence ^6^AKVSKK^11^ and 3′‐acidic tail were also identified as NLSs which regulate SET nuclear import and export.^6^, ^26^, ^27^ As subcellular location directly affects the biofunctions of SET, cells have developed multiple regulatory mechanisms to precisely control its distribution.

## 4 PHYSIOLOGICAL FUNCTIONS OF ONCOPROTEIN SET

**Figure 2:**
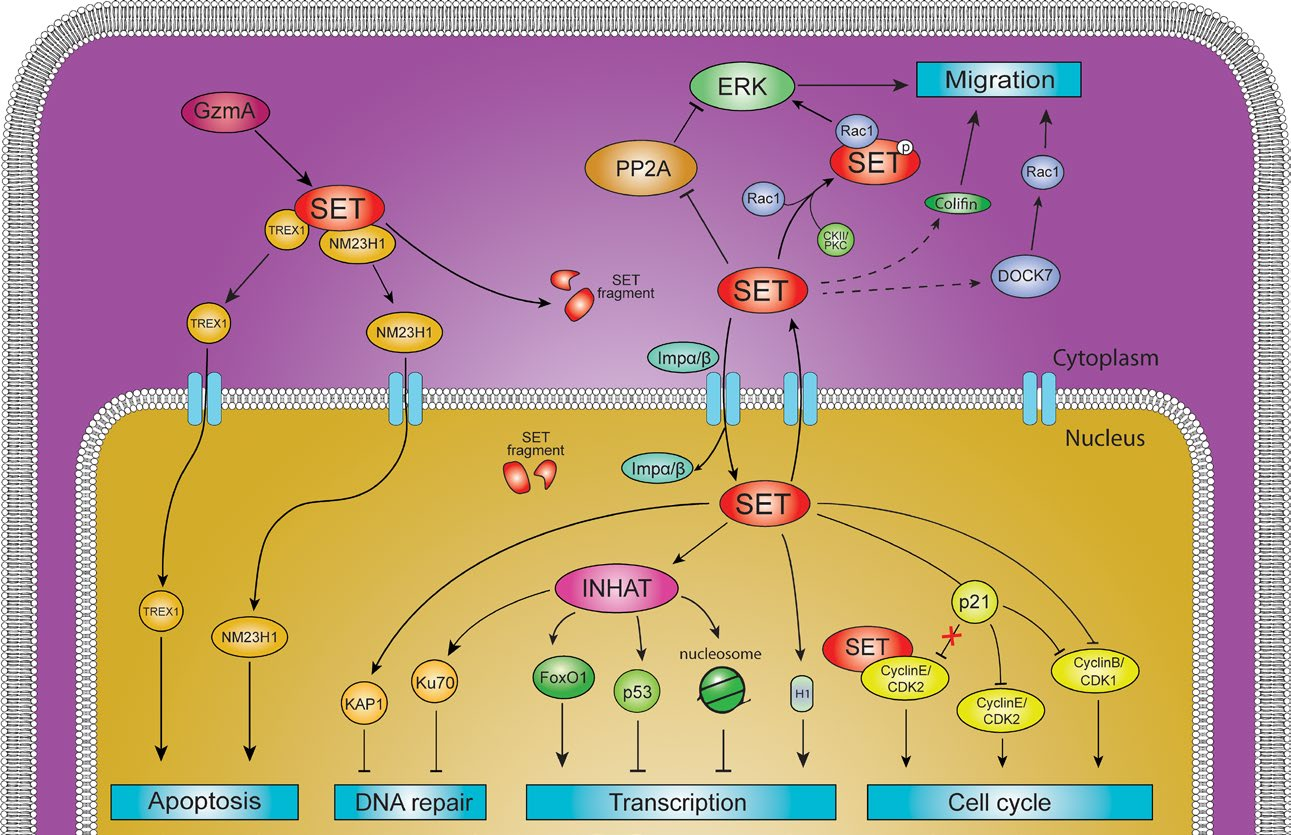
A schematic overview of physiological functions of SET and its interacting proteins. The oncoprotein SET participates in multiple cellular processes, including cell migration, cell cycle, apoptosis, gene transcription, and DNA repair. The SET protein regulates functions of a subset of proteins which are involved in each process. The interacting network among those proteins is shown here. The proteins interacting with SET are indicated as self‐explanatory symbols. The black line represents a direct interaction between SET and proteins, whereas dot line represents the unknown or indirect interaction between them.

The physiological and pathological functions of SET have been widely and extensively studied. SET protein actively participates in numerous cellular processes (Figure 2) and plays an essential role in cell survival and proliferation.

### 4.1 Cell cycle

The process of cell division is precisely regulated by cyclin‐dependent kinases (CDKs) and cyclin complexes.^28^ p21^Cip1^ is known as a strong inhibitor of CDK. SET protein interacts with p21^Cip1^ and specifically modulates its inhibitory effect on the activity of cyclin E‐CDK2 complex that is necessary for G1/S transition. SET also can bind to cyclin E‐CDK2 regardless of the presence of p21^Cip1^.^29^ Cyclin B‐CDK1 complex is essential for G2/M phase transition and phosphorylation of nuclear substrates which are necessary for mitotic onset. Reportedly, the acidic sequence DEDDDE (aa 260‐265) of SET can bind to and inhibit cyclin B‐CDK1. p21^Cip1^ and SET cooperates in the inhibition of cyclin B‐CDK1 activity. Overexpression of SET blocks the cell cycle at the G2/M transition in COS and HCT116 cells.^29^, ^30^ Overall SET reverses the inhibitory effect of p21^Cip1^ on cyclin E‐CDK2 as a positive regulator of G1/S, while SET also takes part in blocking cyclin B‐CDK1 during S phase and G2, avoiding a premature activation of these complexes.^30^

The tumor suppressor p53 functions as a transcriptional factor and regulates numerous RNA Pol II‐transcribed genes, such as WAF1/Cip1 encoding p21^Cip1^. p53 activates expression of p21^Cip1^ protein resulting in inhibition of CDK complexes and cell cycle arrest at the G1/S phase.^31^ SET protein can directly bind to p53 and suppresses p53 activation by acetylation, therefore, promoting G1/S transition during the cell cycle.^20^

### 4.2 PP2A inhibition

PP2A is a serine/threonine phosphatase in eukaryotic cells, and extensively involved in various cellular processes, including protein synthesis, signaling transduction, cell cycle determination, apoptosis, metabolism, and stress response.^32^, ^33^, ^34^, ^35^ As a major heterotrimeric phosphatase PP2A contains a catalytic C subunit (PP2Ac), the scaffold A subunit and the regulatory B subunit. SET protein directly interacts with PP2Ac through its domain (aa 25‐119) and inhibits the phosphatase activity of PP2A. By interfering with the PP2A‐mediated dephosphorylation, SET abolishes the tumor suppressor functions of PP2A and activates oncogenic signaling such as Akt, Erk, c‐Jun, c‐Myc, and β‐catenine to promote the survival and progression of cancer cells.^15^, ^33^, ^35^, ^36^, ^37^

### 4.3 Cell migration and metastasis

Cell migration comprises multiple steps including cell polarization, membrane protrusion and tail retraction, adhesion formation, and directional translocation of the cell body.^38^ Intracellular signaling components, such as small GTPases of the Ras, Rab, Arf, and Rho, families cooperate in the cell migration cycle.^39^ It is well‐known that Rho GTPase Rac1 controls cell adhesion, spreading, and migration.^40^, ^41^ In vivo and in vitro assays have confirmed that Rac1 associates with SET protein. Upon activation of Rac1 in the cytosol, the nucleic protein SET translocates to the plasma membrane and locally amplifies kinase‐mediated signaling. The whole process depends on SET phosphorylation, monomerization, and binding to Rac1. Simultaneously, SET inhibits PP2A, which negatively regulates Rac1 signaling. The SET‐Rac1 complex subsequently cooperates to induce cell motility.^26^ In Esophageal Squamous Cell Carcinoma (ESCC), ectopic expression of SET promotes expression of a subset of genes related to directional migration, including ROCK7 and Cofilin. Cofilin associates with cytoskeleton reorganization. SET‐induced cell motility is impaired by ROCK7 and/or Cofilin knockdown. Additionally, SET epigenetically represses miR‐30c that serves as a direct regulator of the crosstalk between ROCK7/RAC1 and cofilin.^42^ SET protein also forms the SET complex together with protein pp32, HMGB‐2, Ape‐1, Trex 1, and NM23H1.^23^ NM23H1 is a well‐known DNase and tumor metastasis suppressor.^43^ SET is able to bind to NM23H1 and inhibits its activity to break DNA to single strand nicks. Proteolytic cleavage or antagonism of SET will reverse suppression of NM23H1, resulting in anti‐metastatic activity or the caspase‐independent apoptosis.^25^

### 4.4 Gene transcription

In the eukaryotic cells, the nucleosome is assembled from a 147‐bp‐long DNA chain, a histone octamer, and a histone linker. Each octamer contains pairs of H2A, H2B, H3, H4 histones, and the linker histone H1 further compacts the nucleosome into more condensed chromatin arrays. DNA on the chromatin is inaccessible; thus, altering the chromatin structure is necessary before the initiation of replication or transcription. Histone modifiers, chromatin remodeling complexes, and histone chaperones are important factors in regulation of chromatin accessibility.^44^ Bioinformatics has revealed that the most transcriptionally active *cis*‐elements, or transcriptionally poised genes are depleted of both nucleosome and histone H1.^45^, ^46^ Histone chaperones are responsible for nucleosome assembling/disassembling and histone variant exchange, and directly involved in gene transcription.^44^ SET is a member of the nucleosome assembly protein family. Kato et al^46^, ^47^ demonstrated that SET stimulates or represses the expression of various genes and the stimulatory effect depends on its histone chaperone activity additively with histone acetylation. SET is capable of interacting with core histones independent of histone acetylation status. SET remodels chromatin structure to facilitate DNA recognition by transcription factors or Pol II for transcriptional initiation. The linker histone H1 takes part in the formation and stabilization of extensively folded chromatin structures that create an inaccessible barrier to the human genome. The association of the linker histone with chromatin inversely correlates with the transcriptionally active gene regions. SET can evict linker histone H1 from the nucleosome, therefore overcoming H1‐induced gene repression.^45^, ^48^ SET‐α shows a lower histone H1 chaperone activity than SET‐β due to isoelectric differences in its N‐terminal region. Moreover, the N‐terminal region of SET‐α directly self‐binds to the C‐terminal region by intramolecular interaction, and therefore regulates the subtype composition‐dependent histone H1 chaperone activity of the SET‐α dimer.^45^

Acetylation of histones by p300/CBP and PCAF is considered to be a critical step in transcriptional activation. Seo et al^49^ discovered the INHAT complex which contains SET‐α/β and pp32 as subunits. The glutamic and aspartic acid (E/D) rich C‐terminal regions of SET‐α (aa 236‐278) and SET‐β (aa 223‐264) have been identified as the inhibitor domain (INHAT domain). The INHAT domain can bind to lysine residues at histone N‐terminal tails and “masks” histones from histone acetyltransferases (HATs), therefore indirectly inhibiting HAT activity of p300/CBP.^49^, ^50^ Schneider et al^51^ found SET specifically binds to the unmodified N‐terminal tail of histone H3 in HeLa cells. Phosphorylation (S10) and acetylation (K9, K14, K18, and K23) of the N‐terminus of H3 attenuates the effectiveness of INHAT. Similarly, Saavedra et al^52^ clarified that SET regulates the acetylation level of newly synthesized histone H4. SET specifically targets the hypoacetylated and unacetylated histones, but not hyperacetylated histones, thus keeping histones at low acetylation levels, which are generally associated with heterochromatin and transcription silencing. The overexpression of SET also inhibits demethylation of ectopically methylated DNA.^53^ The INHAT domain of SET has also been found to interact with lysine residues of different proteins. Kim et al reported that SET can bind to C‐terminal hypoacetylated lysine residues of p53 and inhibit p300/CBP and PCAF‐mediated p53 acetylation, thus inducing transcriptional repression on p53 target genes. Consequently, SET blocks both p53‐mediated cell cycle arrest and apoptosis in response to cellular stress.^20^, ^54^ Forkhead family transcription factor, FoxO1, is an important regulator of activation of various target genes, affecting cell metabolism, cell survival, and apoptosis. FoxO1 is acetylated by p300/CBP‐mediated acetylation that reduces its DNA binding affinity. Chae et al^55^ found the INHAT domain of SET is able to bind to FoxO1 and inhibits its acetylation, resulting in upregulated transcriptional activity.

SET is also an important regulator of hormone receptor‐mediated gene expression. SET interacts with the glucocorticoid receptor (GR), thyroid hormone receptor (TR), and estrogen receptor (ER) through its earmuff and INHAT domains.^56^, ^57^ In ERα‐positive MCF7 cells, overexpression of SET inhibited p300‐mediated acetylation of histone and ER, resulting in repression of estrogen‐induced gene expression in a dose‐dependent manner.^56^, ^58^ However, this observation conflicts with the fact that SET protein is highly expressed in ER positive breast cancer cells whose growth relies on hormone‐induced gene expression.^15^ Overall, SET has positively or negatively regulated the expression of numerous cellular genes in a gene‐specific manner. The precise mechanisms in gene transcription remain elusive and need to be further investigated.

### 4.5 DNA repair

In cancer cells, oxidative stress typically occurs and is associated with energetic and redox balance alterations. Oxidative stress can cause chromosomal aberrations, DNA damage, and mutations, which contribute significantly to tumor development and progression.^59^, ^60^ SET accumulation has also been reported in cancers and Alzheimer's disease.^6^ The functional role of SET in DNA repair remains unclear. In HEK293T cells, SET protects cells from mild oxidative stress by promoting expression of genes encoding cell antioxidant defense proteins, such as PRDX2, PRDX6, SOD2, TXN, UCP2, and UCP3.^22^ Kalousi et al^61^ demonstrated that the SET protein regulates DNA repair by acting as a repressor of homologous recombination (HR) and DNA damage response (DDR). SET is recruited in the laser‐induced breaks and reduces the protein level of γH2A.X. Additionally, SET protein can bind to KAP1, which recruits methyltransferase SETDB1 for tri‐methylation of histone H3 at Lys‐9 (H3K9me3) and heterochromatin protein 1 (HP1). This SET/KAP1/HP1/H3K9me3 association on the DNA breaks leads to chromatin retention and creates a repressive environment for DNA repair. Kim et al^21^ found that the recruitment of heterodimer Ku70/80 is required as an initial step for the nonhomologous end joining (NHEJ) DNA DSB repair pathway. Lysine residues of Ku70 are targets of acetylation by CBP/p300 and PCAF. The C‐terminal of Ku70 contains 3 lysine acetylation sites (K539, K542, and K544) that can bind to the INHAT domain of SET protein. Under normal conditions, SET interacts with Ku70/80 in the nucleus. Once DNA damage occurs, Ku70/80 dissociates from SET, and resumes the acetylated state by CBP/p300, thereby allowing these proteins to be recruited to DSB sites to initiate DNA repair or BAX‐mediated apoptosis. However, abnormal SET overexpression disrupts the SET and Ku70 protein association pattern, thereby inhibiting DNA repair and Ku70 acetylation‐mediated apoptosis, causing the accumulation of damaged DNA inside cells which eventually leads to mutations and carcinogenesis.

## 5 TRANSCRIPTIONAL AND POST‐TRANSLATIONAL REGULATION OF SET

**Figure 3:**
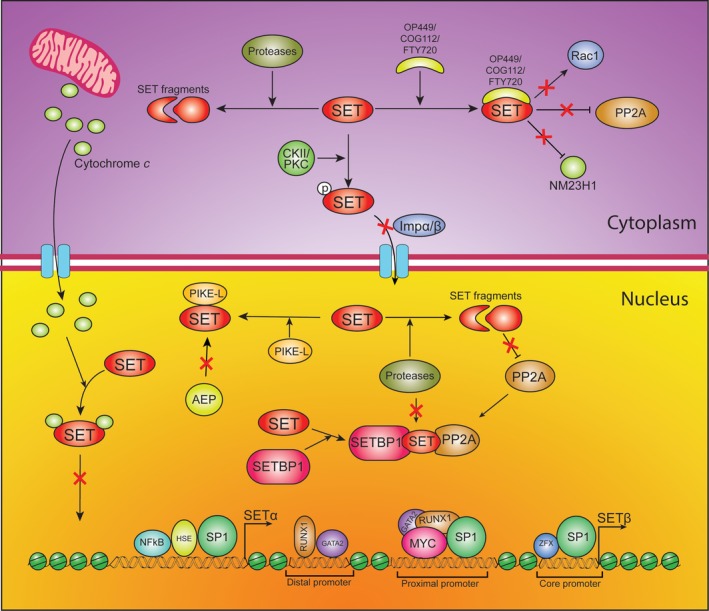
Transcriptional and post‐translational regulation of the SET protein. The expression of SET‐α/β is strictly regulated at both transcriptional and post‐translational levels. The transcriptional activation of SET‐α/β is determined by alternative promoters which contain various *cis*‐regulatory motifs corresponding different transcriptional factors, such as NFκB, SP1, MYC, RUNX1, GATA2, and ZFX. They form the transcriptional complex to precisely initiate the transcription of SET‐α/β gene. Phosphorylation and proteolytic cleavage of SET protein are the main regulatory mechanisms at post‐translational level. Phosphorylation at the site serine 9 affects SET protein subcellular location and enhances its association with PP2A and Rac1 in the cytoplasm. Conversely, the PP2A suppression by the SET protein is significantly impaired by proteolytic cleavage. Additionally, SET‐binding proteins (SETBP1, PIKE‐L, cytochrome *c*) and inhibitors (FTY720, COG112 and OP449) are able to bind to SET protein and regulate its biofunctions.

Due to essential roles in cellular processes, cells establish multiple regulatory mechanisms to precisely modulate the expression, form, and distribution of SET that closely correlates with its physiological function (Figure 3).

### 5.1 Transcriptional regulation

It was found that in rapidly dividing cells SET level is high, whereas its expression is low in quiescent cells^18^. SET overexpression increases significantly as cancer progression occurs. Despite the prognostic impact of SET overexpression in tumorigenesis and metastasis, little is known about the mechanisms involved in the transcriptional regulation of this oncogene. SET‐β is more widely and relatively constantly expressed compared with SET‐α that varies among cell types.^3^ The upstream region of SET‐α promoter contains several putative regulatory elements for binding factors, such as HSE, SP1, and NFκB. Reportedly, recruitment of SP1 and NFκB is necessary for SET‐α gene expression as the gene promoter does not have typical TATA and CAAT boxes.^62^, ^63^ The SET‐β promoter is also TATA‐less and G/C‐rich. There are 2 significant promoter regions in AML harboring different DNA motifs for transcription factors (TFs), for instance, RUNX1 and GATA2 at the distal promoter region (−932/−699 bp), SP1 and MYC at the proximal promoter region (−318 bp/TSS). MYC, SP1, RUNX1, and GATA2 form a multi‐protein transcriptional complex that regulates the transcriptional activation of SET‐β. Deletion of any DNA motif of these TFs results in a significant decrease in the SET expression level.^64^ These TFs are usually overexpressed in cancer cells, thereby enhancing the transcription of SET. Siliang et al found that there are 4 zinc finger and X‐linked factor (ZFX) binding sites in the proximal promoter region that are critical for SET‐β transactivation in both HEK293 and HeLa cells.^65^ Multiple Sp1 binding sites are found in both SET‐α and SET‐β promoter regions, but their effects on each isoform transcription are different. In HEK 293, HeLa, AML, HL‐60, and HEL cells Sp1 positively contributes to SET‐α gene transcription in vivo while Sp1 knockdown leads to reduction in SET‐α mRNA level, whereas Sp1 deficiency has no effect on SET‐β transcription.^63^ Furthermore, reporter assays confirmed that Sp1 overexpression failed to impact on SET‐β promoter activity indicating the distinct transcriptional regulation between the 2 isoform transcripts.

### 5.2 Protein modification and proteolytic cleavage

SET protein is primarily phosphorylated on serine residues.^2^ Additionally SET protein also can be acetylated and glycosylated by TLL8. Phosphorylation at Serine 9 (S9) is well understood. S9 is nested in the center of the NLS sequence ^6^AKVSKK^11^. In neuron cells, casein kinase (CK) II‐mediated phosphorylation at S9 interferes with the formation of the SET/imp3α/impβ complex, and thus inhibits SET nuclear import and induces cytoplasmic retention.^6^, ^24^ Other studies have reported that SET undergoes PI3Kγ‐mediated S9 and S24 phosphorylation by PKC that controls SET nuclear export and, hence, regulates PP2A inhibition.^6^

I2PP2A is a truncated version of the SET protein which indicates that proteolytic cleavage is an important way to regulate its distribution and function. Overexpression experiments in HeLa cells showed that full‐length SET α/β proteins localize in the nucleus, but C‐terminally truncated SET‐α proteins mostly accumulated in the nucleus while C‐terminal truncated SET‐β proteins were localized mainly in the cell cytosol with trace amount in the nucleus.^3^ In neuron cells, camptothecin treatment induces gradual loss of full‐length SET from nuclear fractions. Concurrently, there is increased expression of a smaller cleaved fragment of SET in the cytosol.^24^ In the immune response, during CTL attack, Granzyme A (GzmA) cleaves SET‐β protein at K176, resulting in the caspase‐independent apoptosis.^25^ In 2008, Liu et al identified a lysosomal protease known as asparagine endopeptidase (AEP) that is activated under acidic condition (pH = 6). AEP cleaves SET‐β protein at N175 in the cytosol, thus freeing NM23H1 to induce DNA nicking, resulting in apoptosis of neuronal cells.^66^ Both GzmA and AEP express in a cell type‐specific manner, whereas the cleavage of SET happens in nearly all cell types. The protease(s) responsible for SET cleavage remain unclear and require to be further investigation.

### 5.3 SET‐binding proteins

In order to uncover the possible mechanism of SET‐related leukemogenesis and tumorigenesis, Minakuch et al^67^ in 2001 isolated and identified SET‐binding protein 1 (SETBP1) as the cellular counterpart of SET. SETBP1 gene encodes a 170 kD protein and is located on chromosome 18q21.1. SETBP1 contains a specific SET‐binding domain, Ski homologous region, 6 PEST sequences, and 3 NLS motifs. Indirect immunofluorescent staining on HeLa and HOS cells showed that SETBP1 predominantly locates in the nucleus. The specific interacting domain of SET (aa182‐223) with SETBP1 is located downstream of the SET‐PP2A binding site (aa 17‐196), and the SETBP1‐SET interaction in the nucleus is stronger than the SET‐PP2A interaction. Cristóbal et al^68^ in 2010 first described SETBP1 gene translocation and overexpression in patients with acute myeloid leukemia. This SETBP1 overexpression protects SET from protease cleavage, therefore increasing the PP2A inhibition and promoting proliferation of the leukemic cells. Phosphatidylinositol 3‐kinase enhancer (PIKE) is a brain‐specific nuclear GTPase. PIKE‐L is 1 of 3 isoforms transcribed and spliced from the CENTG1 gene encoding PIKE. In neurons, PIKE‐L interacts with various transmembrane receptors to trigger PI3K activation.^69^, ^70^ Further studies have clarified that PIKE‐L interacts with SET both in vitro and in vivo and protects SET from cleavage by AEP. PIKE‐L is present in both the nucleus and the cytoplasm. System truncation assay has elicited that the domain of SET (77‐127) region is required for the interaction with PIKE‐L.^66^ De la Rosa's laboratory showed that cytochrome *c* translocates and interacts competitively with SET in the nucleus in response to DNA damage. Cytochrome *c* blocks the histone binding domains of SET and inhibits its histone chaperone activity.^71^ Overall, SET‐binding proteins can selectively impede either PP2A inhibition or histone chaperone activity of SET. These findings may subsequently facilitate the development of new and less toxic drugs to silence the specific oncogenic effect of SET function.

## 6 SET AS A NEW ANTICANCER DRUG TARGET

SET plays a vital role in promoting tumorigenesis, metastasis, and development of therapeutic drug resistance and, SET is therefore, a potential biomarker that predicts drug sensitivity and a therapeutic target to enhance current anticancer treatments. Several SET antagonists have been developed and extensively evaluated for cancer therapy. FTY720 (fingolimod) is a sphingosine analog used as an immunosuppressant in multiple sclerosis patients.^16^ FTY720 can reactivate PP2A by the disruption of SET‐PP2Ac interactions, resulting in significant anticancer activity. The anticancer effect of FTY720 has been shown in many hematologic and solid malignancies. FTY720 treatment also inhibits epithelial‐to‐mesenchymal transition (EMT) by affecting SET/PP2A/c‐Myc/NDRG1/Snail signaling, and restore sensitivity to standard treatments such as cisplatin in lung cancer cells, and imatinib in chronic myeloid leukemia cells harboring resistant mutations.^16^, ^72^, ^73^ COG112 is a specific cell‐penetrating peptide. It was designed by fusing a protein transduction domain derived from the *Drosophila* antennapedia protein to the peptide COG133 which is mimetic peptide created from amino acid residues 133‐149 of the Apolipoprotein E (apoE) holoprotein.^74^ COG112 inhibits inflammatory response in Colitis by suppression of NF‐κB signaling and proinflammatory cytokine expression. Later Christensen et al found that COG112 is able to bind to SET expressed in immune cells.^74^ The COG112‐SET interaction inhibits the formation of the SET/PP2A‐c complex, thus increasing cellular PP2A and NM23H1 activity levels. COG112 can also prevent the Rac1‐SET complex formation resulting in the inhibition of migration and invasion.^8^, ^75^, ^76^, ^77^ OP449 derived from COG112 peptide also interacts with SET and antagonizes SET's inhibition of PP2A. OP449 not only effectively reduces SET's inhibition of PP2A, but also has selective cytotoxic properties for leukemic cells.^12^, ^15^ Further studies confirmed antagonism of SET using OP449 synergistically enhances the efficacy of tyrosine kinase inhibitors and overcomes drug resistance in malignancies by combined treatment.^78^, ^79^

## 7 CONCLUSIONS

Accumulating evidence is demonstrating that oncoprotein SET participates in multiple cellular processes, such as cell cycle, cell migration, apoptosis, gene transcription, and DNA repair. High levels of SET expression have been found to correlate with cancer occurrence, metastases, and prognosis. Therefore, SET has received increasing attention from scientists as a potential target for cancer therapy. Structure‐function studies have shown that SET protein interacts with a number of proteins via different domains (Table 1) leading to multiple biofunctions. Due to a critical role in cell survival and growth, cells precisely regulate the expression and activity of SET at both transcriptional and posttranslational levels. Several SET inhibitors are currently being developed and evaluated and appear to have robust anticancer activity. Efforts to fully understand the exact mechanisms of SET in cancer will facilitate the development of new or improved antagonists for cancer therapy.

**Table 1:** Characterization of known SET‐interacting proteins

Interacting proteins	Interaction domain(s)		Functions and references
	Proteins	SET	
AEP	–	–	Apoptosis induction^66^
ApoE	130‐149	C‐terminal	Inhibition of SET^80^
Cyclin B‐CDK1	–	225‐277/260‐265	Cell cycle regulation^30^
Cyclin E‐CDK2	–	–	Cell cycle regulation^29^
Cytochrome *c*	Heme crevice domain	Lower earmuff domain	Inhibition of SET^81^
Estrogen Receptor α	180‐303	133‐225	Transactivation suppression^56^
FoxO1	C‐terminal/lysine	INHAT domain	Transcription activation^55^
Granzyme A	–	–	Apoptosis induction^25^
Histone H1	C‐terminal	NAP domain	Transcription activation^46^, ^47^
Histone H2A/H2B	–	NAP domain	Transcription activation^82^
Histone H3	C‐terminal	INHAT domain	Transcription suppression^49^
Histone H3	–	NAP domain	Transcription activation^83^
Histone H4	C‐terminal	INHAT domain	Transcription surpression^52^
Histone H4	–	NAP domain	Transcription activation^83^
KAP1	–	–	DNA repair repression^61^
Ku70	C‐terminal	INHAT domain	DNA repair repression^21^
MLL	N‐terminal	C‐terminal	Transcription activation^84^
p21^Cip1^	140‐144/156‐164	81‐180/181‐277	Cell cycle regulation^30^
p53	C‐terminal	INHAT domain	Transcription suppression^20^
PIKE‐L	1‐384	77‐127	Protection of SET from cleavage^66^
PP2A	PP2Ac	17‐196	Inhibition of PP2A activity^27^
Rac1	C‐terminal	INHAT domain	Cell migration regulaton^26^
SETBP1	1238‐1434	182‐223	Protection of SET from cleavage^67^

– Not determined.

## CONFLICT OF INTEREST

None declared.
